# Nanoplastic-Induced Developmental Toxicity in Ascidians: Comparative Analysis of Chorionated and Dechorionated *Phallusia mammillata* Embryos

**DOI:** 10.3390/jox15010010

**Published:** 2025-01-10

**Authors:** Maria Concetta Eliso, Ilaria Corsi, Antonietta Spagnuolo, Rémi Dumollard

**Affiliations:** 1 Department of Physical, Earth and Environmental Sciences, University of Siena, 53100 Siena, Italy; ilaria.corsi@unisi.it; 2 Department of Biology and Evolution of Marine Organisms, Stazione Zoologica Anton Dohrn, 80121 Naples, Italy; antonietta.spagnuolo@szn.it; 3 Laboratoire de Biologie du Développement (LBDV), Institut de la Mer de Villefranche (IMEV), Sorbonne Université, Centre National de la Recherche Scientifique (CNRS), 06230 Villefranche-sur-Mer, France; remi.dumollard@imev-mer.fr

**Keywords:** nanoplastics, polystyrene nanoparticles, PS-NH_2_, *Phallusia mammillata*, embryotoxicity, oxidative stress, neurodevelopment, genotoxicity, morphometric analysis

## Abstract

Nanoplastics pose a growing threat to marine ecosystems, particularly affecting the early developmental stages of marine organisms. This study investigates the effects of amino-modified polystyrene nanoparticles (PS-NH_2_, 50 nm) on the embryonic development of *Phallusia mammillata*, a model ascidian species. Both chorionated and dechorionated embryos were exposed to increasing concentrations of PS-NH_2_ so morphological alterations could be assessed with a high-content analysis of the phenotypes and genotoxicity. PS-NH_2_ induced the same morphological alterations in both chorionated and dechorionated embryos, with dechorionated embryos being more sensitive (EC_50_ = 3.0 μg mL^−1^) than chorionated ones (EC_50_ = 6.26 μg mL^−1^). Interestingly, results from the morphological analysis showed two concentration-dependent mechanisms of action: (i) at concentrations near the EC_50_, neurodevelopmental abnormalities resembling the ones induced by exposure to known endocrine disruptors (EDs) were observed, and (ii) at higher concentrations (15 μg mL^−1^ and 7.5 μg mL^−1^ for chorionated and dechorionated embryos, respectively), a nonspecific toxicity was evident, likely due to general oxidative stress. The phenotypes resulting from the PS-NH_2_ treatment were not related to DNA damage, as revealed by a genotoxicity assay performed on neurula embryos. Our data suggest that PS-NH_2_-induced toxicity is primarily mediated through oxidative stress, probably triggered by interactions between the positive charges of the PS NPs and the negative charges on the cell membranes. The lack of a protective chorion further exacerbated these effects, highlighting its role in mitigating/protecting against NP-induced damage.

## 1. Introduction

Plastic pollution is recognized to be a threat of global concern for the marine environment. Approximately 5000 tons of plastic waste was released into the environment globally in 1950–2015, and, as plastic litter ages, it breaks down, forming microplastics (MPs, <5 mm) and nanoplastics (<100 nm), via weathering, sunlight radiation and biodegradation processes [1,2,3,4,5,6]. The formation of smaller particles leads to a modification of the physical–chemical properties, surface area and size, and, once below 1 µm in size, nanoplastics can have high reactivity and biotoxicity Although environmental concentrations of nanoplastics are still unknown, it is well accepted that they are ubiquitous, as recently found in the surface water of the North Atlantic Gyre [7] and in the West Mediterranean Sea [8]. They indeed represent a serious hazard for marine species, in particular in marine coastal areas [9,10,11]. On these grounds, in recent years, several studies have focused on exploring the impact of nanoplastics on marine organisms by using, among a wide variety of polymer compositions, polystyrene nanoparticles (PS NPs) as a proxy for nanoplastic [12] and references therein]. The studies showed that these small particles can affect marine phyto- and zooplankton, resulting in oxidative stress and feeding disruption, among other sub-lethal effects [13,14,15,16,17,18,19,20,21,22,23,24,25]. Recently, the effects of PS NPs (PS-NH_2_, 50 nm) on chorionated *Ciona robusta* embryos have been investigated, revealing impairment in trunk and palps morphogenesis and oxidative stress in the larvae [18]. Furthermore, the development of an Adverse Outcome Pathway (AOP) exploiting transcriptomic data and sub-lethal endpoints suggested that a Molecular Initiating Event (MIE), the adhesion of PS NPs to the ascidian chorion, an envelope that surrounds the egg and the developing embryo until the hatching of the tadpole larva [26,27,28,29], may act as a physico-chemical barrier against pollutants [19].

Ascidians, which are abundant components in marine meso-zooplankton communities and have an invertebrate embryo that is closely related to vertebrates [30,31,32], represent ideal models for ecotoxicity studies aimed at exploring the effects of a large variety of physical and chemical stressors [33,34]. Here, we investigate the effect of amino-modified PS NPs (PS-NH_2_, 50 nm) on the embryonic development of another ascidian species, *Phallusia mammillata*. This marine invertebrate is found exclusively in the Northeast Atlantic and the Mediterranean and it is an emerging ascidian model for developmental and molecular biology studies (e.g., live-cell imaging) since its eggs and embryos are completely transparent [35,36,37,38]. In addition, genomic and transcriptomic resources are available, thus facilitating functional gene studies (https://www.aniseed.cnrs.fr/; accessed on 20 November 2022). In the past years, *Phallusia* has been used in ecotoxicology for the testing of the embryotoxicity of several Contaminants of Emerging Concerns (CECs), at both morphological and molecular levels [39,40,41,42], and, more recently, high-content analyses of the morphological alterations of *Phallusia* larvae have been exploited to determine the phenotypic signature of different classes of chemicals such as Endocrine Disruptors (EDs), Nuclear Receptors (NRs), ligands and genotoxic and cytotoxic compounds [43]. In this work, the PS-NH_2_ was tested on chorionated *Phallusia* embryos, resembling the natural exposure scenarios, and on dechorionated embryos, to evaluate the effects of direct contact with the growing embryos and unravel the potential protective/mitigation role against NP exposure. Then, high-content-based morphometric analysis of *Phallusia* larvae allowed us to quantify morphological malformations with neurodevelopmental endpoints. The morphometric analysis was coupled to a genotoxicity assay to explore the possible toxic mechanisms of action.

## 2. Materials and Methods

### 2.1. Amino-Modified PS NPs (PS-NH_2_)

Unlabeled 50 nm amino-modified PS NPs (PS-NH_2_, PA02N, lot: 12839) were purchased from Bangs Laboratories Inc. (Fishers, IN, USA) and received as a 100 mg mL^−1^ stock suspension in deionized water with no added surfactants, according to the manufacturer’s technical sheet. After a brief sonication, as already described in the study of Eliso et al., 2020 [18], intermediate suspensions (10 mg mL^−1^) were prepared in 0.22 μm filtered milli-Q water (mQW) and stored in sterile vials at 4 °C until use. For the embryotoxicity assay, a PS-NH_2_ working suspension (1 mg mL^−1^) was prepared in Natural Sea Water (NSW, salinity 40‰, pH 8) collected from a clean site in the Mediterranean Sea as the exposure media without further sonication. A detailed physico-chemical characterization of PS-NH_2_ behavior in mQW and NSW media is reported in the study of Eliso et al., 2020 [18]. In particular, in mQW, PS-NH_2_ confirmed the primary size (50 nm), and the positive surface charge (+47.5 mV), and showed an optimal dispersion (PDI 0.175 ± 0.04) at different time points. Conversely, in NSW, PS-NH_2_ was found to form large agglomerates (Z-Average of 999.7 ± 54.19 nm) with broader PDI values (>0.400), and a decrease in the ζ-potential values (+7.19 mV) showed the instability of PS NPs in this high-ionic-strength medium [18]. Details on the Fourier Transform Infrared (FTIR) analysis used to determine the structure and composition of the stock suspension are provided in the Supplementary Materials.

### 2.2. Animal and Gametes Collection

Adult specimens of *Phallusia mammillata* were collected in Sète (Hérault, France) and kept at 17 ± 1 °C in circulating natural seawater aquaria. Animals were kept under constant light conditions to avoid uncontrolled spawning of eggs and sperm [44]. The experimental design included two different fertilization protocols: (i) the exposure of embryos with a chorion (chorionated embryos), to mimic the natural exposure condition in the water column; (ii) exposure of embryos without a chorion (dechorionated embryos) according to a protocol already used to screen the toxic effect of several chemicals using the *Phallusia* larval teratogenic assay [42,43]. For the fertilization of chorionated eggs, eggs and sperm were collected separately by dissecting the gonoducts of several hermaphrodite adults. The sperm was stored at 4 °C until use, while oocytes were collected in tissue culture plates and rinsed twice in 0.22 µm filtered NSW, and immediately fertilized by adding diluted sperm (1:100 in NSW) to the egg suspension. After 10 min of incubation, eggs were rinsed 3 times with NSW. For the fertilization of dechorionated eggs, the protocol was the same, except for the presence of the chorion, which was removed from the eggs before fertilization. To remove the chorion, eggs were incubated in a trypsin solution for 2 h and then washed 3 times using NSW supplemented with TAPS buffer (NSW/TAPS; 0.5 mM) according to the protocol described in the study of Dumollard et al. (2017) [35].

### 2.3. Embryotoxicity Assay

About 1 h 30 min post-fertilization (hpf), 60 chorionated embryos (~two-cell stage) were added to 6-well plates containing PS-NH_2_ suspensions in NSW (final volume 6 mL). For the exposure of dechorionated embryos, the 60 embryos were instead transferred at the zygote stage (1-cell stage, around 30 min after fertilization) to avoid the dissociation of 2-cell-stage embryos during the transfer. Both chorionated and dechorionated embryos were exposed to nominal concentrations of PS-NH_2_ as follows: 2–5–7.5–10–15 μg mL^−1^ for chorionated embryos, and 2–3–3.5–5–7.5 μg mL^−1^ for the dechorionated ones. Embryos were then incubated under static conditions in the dark at 18 °C until the hatching larva stage (stage 26) was reached at about 18 hpf. After the incubation period, larvae were fixed in 4% paraformaldehyde (4% PFA, 0.5 M NaCl, PBS; Sigma, St. Louis, MO, USA), washed 3 times in Phosphate-Buffered Saline (PBS 1X) and imaged by transmitted light microscopy (Zeiss Axiovert 200, Jena, Germany) at 10× magnification for the evaluation of the percentage of normal hatched larvae and the morphometric analysis. As described by Gomes and collaborators (2019) [42], a *Phallusia* larva is considered normal when it shows a good general embryo morphology, with a proper trunk and palps formation, as well as tail elongation.

### 2.4. Morphometric Analysis of Larval Development

For the morphometric analysis, bright-field images (Zeiss Axiovert 200) of both chorionated and dechorionated embryos were analyzed with the in-house software Toxicosis8 (Version 1, reference IDDN.FR.001.330013.000.S.P.2018.000.10000, deposited on July 13th 2018; see full description of Toxicosis8 in the study of Gazo et al., 2021 [43]). At least 50 tadpoles were analyzed per treatment. The 5 endpoints quantified were the area of the two pigmented cells (PCs) (Oc/Ot area, μm^2^), the distance between the two PCs (Oc/Ot distance, μm), the length/width ratio of the trunk, the length of the tail and the presence of well-elongated palps. The resulting data were normalized to each respective control treatment (100%) and plotted in radar charts for better comparison of phenotypes between treatments (see [43] for a full description of the morphometric analysis).

### 2.5. Genotoxicity Assay

Genotoxicity testing was performed only on dechorionated embryos to better visualize the fluorescent nuclei within the embryos. Embryos at the neurula stage were fixed with a fixation solution (4% paraformaldehyde, 0.5 M NaCl in PBS) for 1 h at 20 °C on a shaker. By washing them twice with PBS, the fixative was removed. After washing, the samples were incubated in PBS containing 0.1% Triton X-100 and 3% bovine serum albumin (PBSB) for 1 h at 20 °C on a shaker. The embryos were stained with 1 μg mL^−1^ Hoechst in PBSB for 1–2 h at 20 °C on a shaker. Finally, the embryos were washed twice with PBSB and transferred on a glass slide. DNA-stained embryos were imaged using epifluorescence microscopy (Zeiss, Axiovert 200) with a 40×/0.8 NA water objective lens, and DNA aberrations (multinucleated cells, micronuclei or DNA bridges) were scored manually. As was performed in the study of Gazo et al., 2021 [43], the number of embryos hosting a DNA aberration was counted. The total number of embryos scored was at least 150 per condition.

### 2.6. Statistical Analysis

All the statistical analyses were performed using Graphpad Prism (Version 8.0.1, San Diego, CA, USA). All data are expressed as mean ± standard deviation (SD). The median effective concentration (EC_50_), corresponding to a 50% reduction in normal hatched larvae, was calculated using a sigmoidal dose−response model according to the following equation:

y = b + (a − b)/1 + 10^(LogEC50−x)^

where y is the response, b is the response minimum, a is the response maximum, x is the logarithm of effect concentration and EC_50_ is the concentration of effect giving 50% of the maximum effect. Data were normalized to the control mean percentage of larval abnormality using Abbot’s formula:

P = (Pe − Pc/100 − Pc) × 100

where Pc and Pe are the control and the experimental percentages of response, respectively. For the morphometric analysis, in order to eliminate the effect of external factors, we compared and normalized each endpoint with the corresponding value in the control group obtained on the same day. The raw data (not normalized) from this assay were statistically analyzed using the Kruskal–Wallis test followed by Dunn’s post hoc test.

## 3. Results

### 3.1. PS-NH_2_ NPs Alter the Normal Larval Development

**Figure 1:**
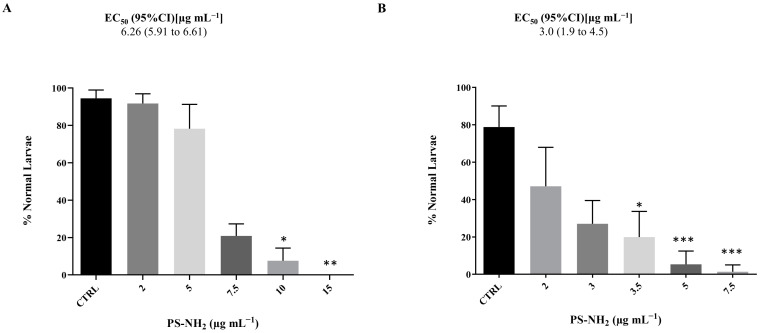
Percentage (%) of normal hatched larvae of *P. mammillata* upon 22 h exposure of chorionated embryos (**A**) and dechorionated embryos (**B**) to PS-NH_2_ in NSW. Bars represent mean ± SD (PS-NH_2_ N = 240). Asterisks indicate values that are significantly different compared to the control (Kruskal–Wallis test, Dunn’s post hoc test (* *p* < 0.05, ** *p* < 0.01, *** *p* < 0.001)). EC_50_ values are shown.

The results of the embryotoxicity assay were evaluated by looking at the normal larval development on both chorionated and dechorionated embryos after an exposure to PS-NH_2_ lasting about 18 h (stage 26). Figure 1 shows a dose-dependent response under both tested conditions, with an increased sensitivity of the dechorionated embryos (EC_50_ = 3.0 µg mL^−1^) compared to chorionated ones (EC_50_ = 6.26 µg mL^−1^).

### 3.2. Quantitative Analysis of Phenotypes

**Figure 2:**
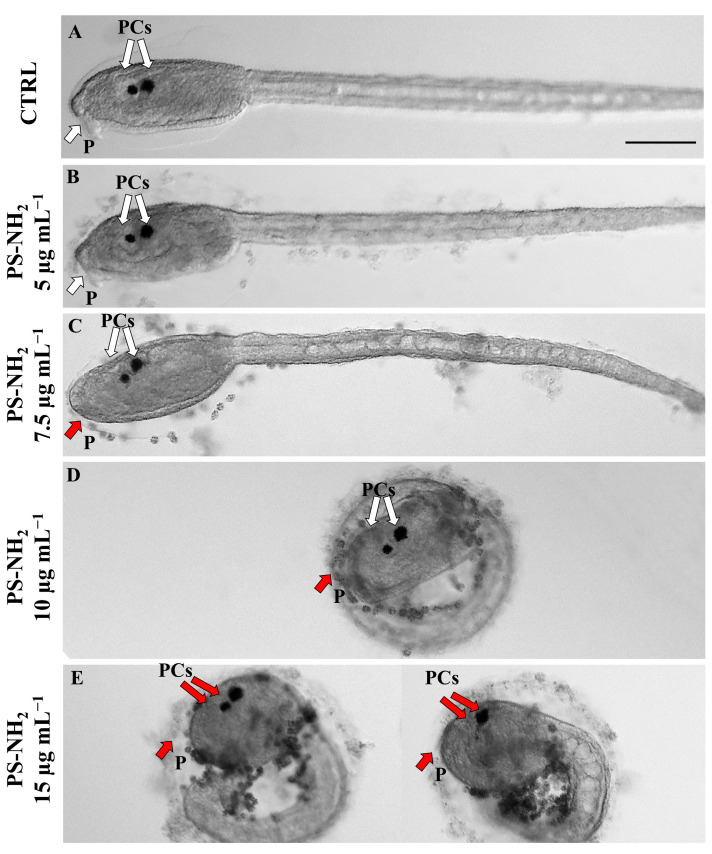
Light microscopy images of *P. mammillata* embryos exposed for 22 h to PS-NH_2_. (**A**–**E**) represent the phenotypes for embryos developed in a chorion: (**A**) control; (**B**) 5 μg mL^−1^; (**C**) 7.5 μg mL^−1^; (**D**) 10 μg mL^−1^; (**E**) 15 μg mL^−1^. White and red arrows represent a good and wrong shape of pigmented cells (PCs) and palps (P), respectively. Scale bar: 100 μm.

In addition to the normal larval development, we also evaluated any phenotypic alterations in developing *P. mammillata* embryos exposed to PS-NH_2_ and then we quantified them using the software Toxicosis8 [43]. Observation under a light microscope indicated that the morphological defects were mainly related to the development of the trunk. As shown in Figure 2B, most chorionated embryos exposed to 5 μg mL^−1^ of PS-NH_2_ showed a generally good morphology of the embryo, comparable to unexposed ones (controls only in NSW) (Figure 2A). At 7.5 μg mL^−1^, we observed a lack of protrusion of the palps in most larvae and, upon exposure to 10 and 15 μg mL^−1^ of PS-NH_2_, the embryos failed to hatch, showing malformations at both the trunk and tail levels. In fact, as shown in Figure 2D,E, the trunk became rounder and the tail shorter compared to the controls (Figure 2D,E).

**Figure 3:**
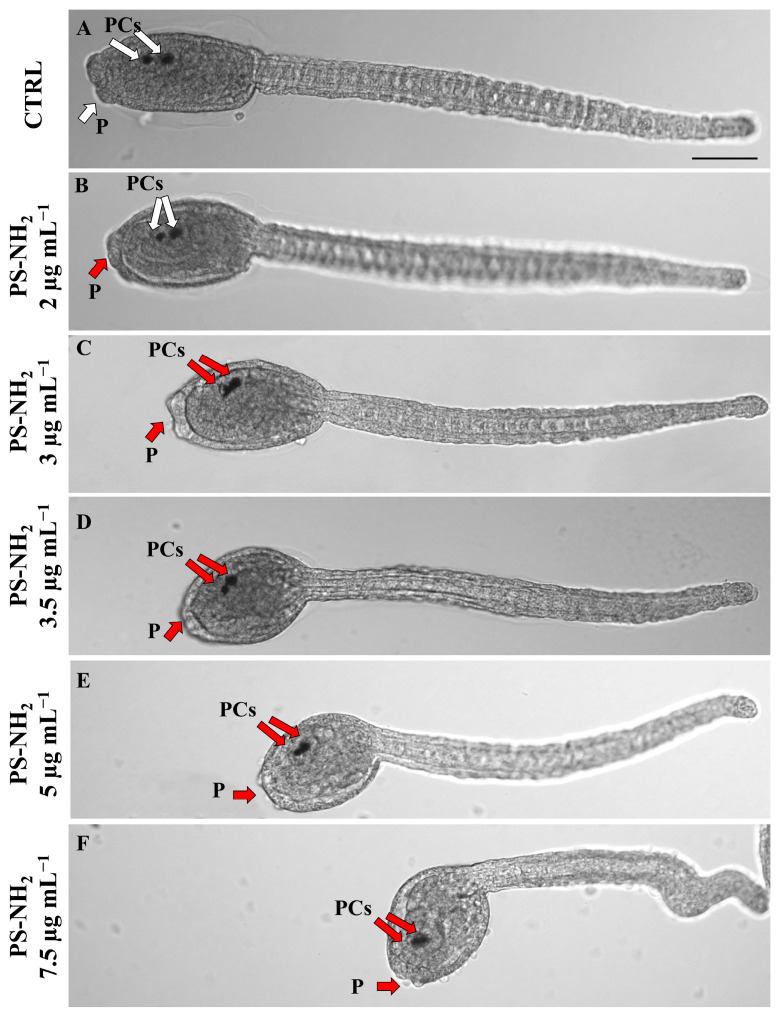
Light microscopy images of *P. mammillata* embryos exposed for 22 h to PS-NH_2_. (**A**–**E**) represent the phenotypes for embryos developed without a chorion: (**A**) control; (**B**) 2 μg mL^−1^; (**C**) 3 μg mL^−1^; (**D**) 3.5 μg mL^−1^; (**E**) 5 μg mL^−1^; (**F**) 7.5 μg mL^−1^. White and red arrows represent a good and wrong shape of pigmented cells (PCs) and palps (P), respectively. Scale bar: 100 μm.

The phenotypic alterations observed in the dechorionated embryos were comparable to the morphological defects detected on chorionated embryos, with an increased sensitivity at lower exposure concentrations. In fact, a rounder trunk and an inhibition of palps protrusion were already observable in larvae exposed to 2–3 μg mL^−1^ of PS-NH_2_ (Figure 3A–F). Moreover, the pigmented sensory organs (PCs, composed of the otolith and ocellus) appeared to be fused already in larvae exposed to 3 µg mL^−1^ and upwards, indicating that the movement of the otolith towards the ventral side of the sensory vesicle was impaired (Figure 3C–F) [45].

**Figure 4:**
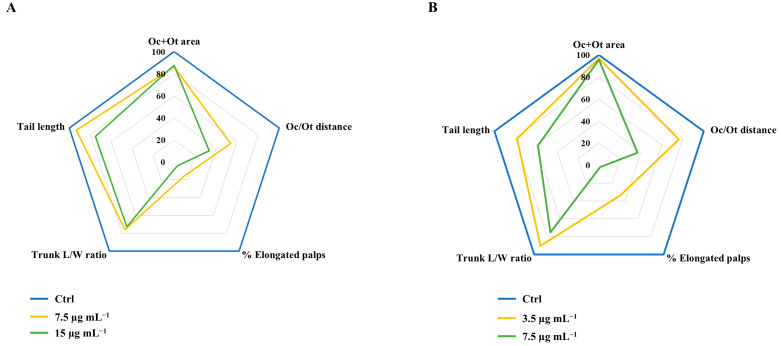
Morphometric analysis of phenotypes induced in *P. mammillata* embryos (**A**) with a chorion and (**B**) without a chorion exposed to PS-NH_2_ (0; 7.5 and 15 μg mL^−1^ for chorionated embryos, 0; 3.5 and 7.5 μg mL^−1^ for dechorionated embryos). The radar charts summarize the following endpoints: ocellus (Oc) + otolith (Ot) area (µm^2^); Oc/Ot distance (µm); percentage of embryos with palps (%); trunk L/W (length/width) ratio; tail length (µm). All measurements were performed at 22 hpf. The values are normalized to the corresponding value of the same parameter in the control (stage 26) and presented as a percentage of the control value. Complete radar charts of all tested concentrations are shown in Figure S3.

Using the Toxicosis8 software, five morphometric endpoints were quantified in chorionated and dechorionated *Phallusia* embryos treated with PS-NH_2_. In chorionated embryos, the most sensitive endpoint was the elongation of the palps, which gradually decreased to 1.8% in larvae exposed to 15 µg mL^−1^, then the Ot-Oc distance (significantly reduced to 36.9%), tail length (reduced to 58.3%) and the trunk L/W ratio (reduced to 75.3%), whereas Oc-Ot area was not affected (Figure 4A, Table 1). Interestingly, dechorionated embryos displayed phenotypes similar to those of chorionated embryos, as illustrated in the radar chart, which shows almost the same order of affected endpoints: palps > Oc-Ot distance > tail > trunk (Figure 4B, Table 2). The only difference is that naked embryos were more susceptible to PS-NH_2_ exposure compared to chorionated ones (compare data at 7.5 µg mL^−1^ in Table 1 and Table 2).

### 3.3. PS-NH_2_ NPs Are Not Genotoxic

**Figure 5:**
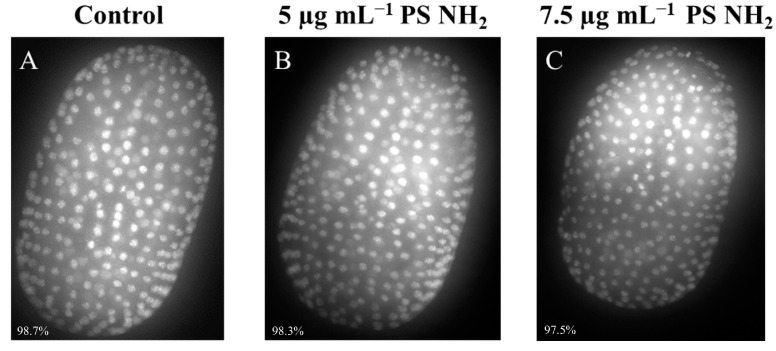
Genotoxicity assay performed analyzing 7 hpf embryos (neurula stage). (**A**) Control embryo (NSW), (**B**) 5 μg mL^−1^; (**C**) 7.5 μg mL^−1^. At the bottom of each image, the percentage of embryos not showing DNA aberrations is reported (N control = 152; 5 μg mL^−1^ = 175; 7.5 μg mL^−1^ = 160.).

Recent data on *Phallusia* embryos indicate that the alteration of palps and trunk elongation are part of the severe malformations associated with DNA damage and genotoxicity [43]. We thus sought to verify whether the phenotypes induced by PS-NH_2_ treatment could also be related to DNA damage, by staining embryo DNA and scoring DNA aberrations in dechorionated embryos at the neurula stage. Figure 5 shows representative images of control and treated embryos (5 and 7.5 µg mL^−1^). In the control cultures, almost all analyzed embryos (98.7%) showed well-aligned nuclei of constant size and tightly packed nuclear DNA without DNA aberrations (Figure 5A). The same results were obtained with treated embryos, with 98.3% and 97.5% of embryos having intact DNA at 5 and 7.5 µg mL^−1^ exposure concentrations, respectively.

## 4. Discussion

Early life stages of marine invertebrate and fish species are sensitive indicators of environmental pollutants including nanoplastics. Here, we investigated the effects of PS-NH_2_ on the development of the ascidian *P. mammillata* by evaluating its impact on embryos, either chorionated or dechorionated, to shed some light on the potential role of the chorion in mitigating the effects of nanoplastics.

Regarding chorionated embryos, the microscopic observations showed morphological alterations mainly at the trunk level; trunks were rounded, and there was a reduction in the protrusion of the palps at exposure concentrations of up to 7.5 μg mL^−1^. Upon exposure to 10 and 15 μg mL^−1^, the embryos failed to hatch, showing malformations at both trunk and tail levels. This phenotype is reminiscent of that previously detected on larvae of another ascidian species, *Ciona robusta* [18], thus suggesting a similar mechanism of action of PS-NH_2_ in *Ciona* and *Phallusia* embryos. Here, we also checked if the direct contact with PS-NH_2_ could have different or even more devastating effects on chorion-deprived developing embryos. Our data indicate that there are no strong phenotypic differences, at the larval stage, between embryos with and without a chorion, except that dechorionated embryos are more sensitive to PS-NH_2_ treatment, on the basis of EC_50_ (3.0 µg mL^−1^ compared to 6.26 µg mL^−1^).

Through the use of the Toxicosis8 software, three neurodevelopmental endpoints (Oc/Ot area, Oc/Ot distance, % of embryos with elongated palps) and two general morphogenesis endpoints (trunk L/W ratio and tail length) were analyzed and the results were compared to previous data obtained in a toxicity screening aimed at finding phenotypic signatures in *Phallusia* embryos associated with xenobiotics [43]. Our study indicates that PS-NH_2_ was able to affect all endpoints analyzed, regardless of the presence or absence of the chorion. Furthermore, the use of this software allowed us to identify two concentration-dependent mechanisms of action: (i) at concentrations near the EC_50_, neurodevelopmental abnormalities, resembling the ones induced by exposure to known endocrine disruptors (EDs), were observed, and (ii) at higher concentrations (15 μg mL^−1^ and 7.5 μg mL^−1^ for chorionated and dechorionated embryos, respectively), a nonspecific toxicity was evident, likely due to general oxidative stress.

Specifically, in the case of concentrations near the EC_50_ (6.26 µg mL^−1^ and 3.0 µg mL^−1^, respectively, for embryos with and without a chorion), the significantly affected endpoints were trunk L/W ratio and tail length, which reflect the general morphology of the embryo, and Oc/Ot distance, which is a marker of the central nervous system. The mechanism of action seems similar since the endpoints were the same regardless of the presence or absence of the chorion. The higher sensitivity of non-chorionated larvae suggests a protective role of the chorion which limits the interaction of the PS-NH_2_ with the embryos. Notably, the phenotypic signatures, such as the trunk L/W ratio and Oc/Ot distance at the lowest doses tested, which characterize *Phallusia* embryos exposed to PS-NH_2_, were very similar to the ones caused by Rifampicin and SR12813, which are PXR agonists [43]. PXR is known to be involved in mechanisms of defense against endogenous and exogenous molecules, regulating the transcription of enzymes and transporters involved in the metabolism and elimination of potentially harmful compounds [46]. In ascidians, this receptor is expressed in the brain vesicles of embryos and larvae and exposure to its agonist induces significant changes in trunk elongation and PC formation [42]. Our findings suggest a possible interaction between PS NPs and PXRs, although we cannot rule out the possibility that the byproducts released by PS-NH_2_ [19], rather than PS-NH_2_, interact with PXR, resulting in the malformations during the embryonic development of ascidians. Actually, as previously shown, styrene monomers and the 2,4-Di-tert-butylphenol (DTBP), which is considered an analog of Bisphenol A (BPA), which is known to interact with PXR in the ascidian *C. robusta*, both leach from the PS-NH_2_ suspension in NSW [19,47,48]. However, the phenotypic signature of PS NPs in *Phallusia* also resembles the one of ERR and RXR ligands [43] and only the measurement of the modulation of the activity of these nuclear receptors by PS-NH_2_ can reveal whether nanoparticles or chemicals leaching from them can affect ascidian nuclear receptors.

On the other hand, the highest concentration of PS-NH_2_ (15 μg mL^−1^ and 7.5 μg mL^−1^ for chorionated and dechorionated embryos, respectively) caused significant effects at the level of all the endpoints studied, except for the Oc/Ot area, suggesting either nonspecific toxicity or DNA damage [43]. However, our study shows that, in PS-NH_2_-treated embryos, the DNA is intact, without any damage, meaning that no genotoxic effects are associated with the exposure to PS-NH_2_ up to 15 μg mL^−1^. The mechanism of toxicity induced by the highest concentration of PS-NH_2_ may be due to the generation of Reactive Oxygen Species (ROS) through positive charges that interact with the negatively charged cell membranes [49]. The elevated levels of ROS may, in turn, cause significant decreases in cell viability and changes in membrane integrity [50]. This is in line with our formulated hypothesis on *Ciona* embryos exposed to PS-NH_2_ [19]. In fact, the initial event leading to the above morphological changes could be the adhesion of PS-NH_2_ around the chorion, potentially causing a general condition of oxidative stress. The influence of positive charge on embryonic development is highlighted in studies showing that negatively charged polystyrene nanoparticles (e.g., PS-COOH) exhibit reduced toxicity during the embryogenesis of the sea urchin *P. lividus* [8] and the ascidian *C. robusta* [21]. These nanoparticles do not affect larval phenotypes or development, likely due to their tendency for rapid sedimentation. The similar phenotype observed in dechorionated embryos suggests that PS-NH_2_ elicits comparable responses in both chorionated and dechorionated embryos, with the observed phenotype likely resulting from direct interaction of the nanoparticles with the developing larvae. From this perspective, it would be very interesting to define the Toxicosis phenotypic signature of oxidative stress and compare it with the phenotypic signature of PS-NH_2_, identified in this study in *Phallusia*, in order to clarify this issue.

## 5. Conclusions

Using high-content analysis of embryonic phenotypes, we established the effects of PS-NH_2_ on the embryogenesis of the ascidian *P. mammillata*. We compared the effects using chorionated (to reproduce the natural conditions) and dechorionated embryos (to ensure the interaction of PS NPs with the embryo), showing that the morphological alterations are the same but the dechorionated embryos have an increased sensitivity, suggesting the protective role of the chorion against PS-NH_2_. The quantitative analysis revealed two different phenotypic signatures for both the conditions tested: (i) the concentrations near the EC_50_ affected trunk L/W ratio, tail length and Oc/Ot distance endpoints, as in the case of PXR agonists; (ii) the highest concentrations of PS-NH_2_ affected several endpoints, suggesting a nonspecific toxicity of PS NPs probably caused by the general stress of the developing organism. Further studies are, however, fundamental to clarify if their toxic effects are due to their adhesion around the growing embryos, or to their internalization within the cells. Moreover, we cannot exclude that the toxic effects are not related to nanoparticles themselves, but to leachates from PS, like styrene monomers and the DTBP [19], which could act directly on naked embryos or by crossing the chorion.

## Figures and Tables

**Table 1:** Quantitative analysis of the morphological aberrations observed after the exposure of *Phallusia mammillata* chorionated embryos to different concentrations of PS-NH_2_. N represents the number of observed embryos at stage 26 (22 hpf). The endpoints analyzed were the ocellus (Oc) + otolith (Ot) area (μm^2^); Oc/Ot distance (μm); percentage of embryos with palps (%); trunk L/W (length/width) ratio; tail length (μm). Data are presented as means ± S.D. and as % compared to the control. Asterisks indicate a significant difference compared to the control (Kruskal–Wallis test and Dunn’s correction, * *p* < 0.05, ** *p* < 0.01, *** *p* < 0.001).

Treatment	N	Oc/Ot Area, μm^2^, Mean ± S.E.	Oc/Ot Area % Compared to the Control	Oc/Ot Distance, μm, Mean ± S.E.	Oc/Ot Distance % Compared to the Control	% Elongated Palps, Means ± S.E.	Elongated Palps % Compared to the Control	Trunk L/W Ratio, Means ± S.E.	Trunk L/W % Compared to the Control	Tail Length, μm, Means ± S.E.	Tail Length % Compared to the Control
Ctrl	143	276.4 ± 60.9	-	18.8 ± 6.7	-	100.0 ± 0.0	-	1.9 ± 0.2	-	589.7 ± 98.7	-
2 µg mL^−1^ PS-NH_2_	85	271.0 ± 64.4	98.0	19.1 ± 6.3	101.3	86.7 ± 8.2	86.7	1.9 ± 0.2	104.1	553.5 ± 94.0	93.9
5 µg mL^−1^ PS-NH_2_	110	274.5± 65.0	99.3	18.3 ± 8.8	97.0	61.8 ± 17.2	61.8	2.0 ± 0.2	105.2	524.4 ± 92.6 ***	88.9
7.5 µg mL^−1^ PS-NH_2_	134	267.9 ± 64.6	96.9	14.4 ± 10.0 **	76.3	33.5 ± 17.9	33.5	1.7 ± 0.4 ***	90.5	461.4 ± 104.7 ***	78.2
10 µg mL^−1^ PS-NH_2_	105	268.8 ± 56.9	97.3	11.6 ± 10.4 ***	61.7	23.2 ± 15.9 *	23.2	1.6 ± 0.3 ***	85.8	436.6 ± 93.5 ***	74.0
15 µg mL^−1^ PS-NH_2_	58	264.9 ± 67.1	95.8	6.9 ± 9.5 ***	36.9	1.8 ± 3.7 ***	1.8	1.4 ± 0.3 ***	75.3	343.6 ± 120.5 ***	58.3

**Table 2:** Quantitative analysis of the morphological aberrations observed after the exposure of *Phallusia mammillata* dechorionated embryos to different concentrations of PS-NH_2_. N represents the number of observed embryos at stage 26 (22 hpf). The endpoints analyzed were the ocellus (Oc) + otolith (Ot) area (μm^2^); Oc/Ot distance (μm); percentage of embryos with palps (%); trunk L/W (length/width) ratio; tail length (μm). Data are presented as means ± S.D. and as % compared to the control. Asterisks indicate a significant difference compared to the control (Kruskal–Wallis test and Dunn’s correction,* *p* < 0.05, ** *p* < 0.01, *** *p* < 0.001).

Treatment	N	Oc/Ot Area, μm^2^, Mean ± S.E.	Oc/Ot Area % Compared to the Control	Oc/Ot Distance, μm, Mean ± S.E.	Oc/Ot Distance % Compared to the Control	% Elongated Palps, Means ± S.E.	Elongated Palps % Compared to the Control	Trunk L/W Ratio, Means ± S.E.	Trunk L/W % Compared to the Control	Tail Length, μm, Means ± S.E.	Tail Length % Compared to the Control
Ctrl	137	294.9.0 ± 64.0	-	23.6 ± 7.7	-	96.3 ± 3.1	-	1.8 ± 0.3	-	577.2 ± 94.6	-
2 µg mL^−1^ PS-NH_2_	122	256.2 ± 107.4	86.9	17.2 ± 10.5 ***	72.9	79.5 ± 19.4	82.6	1.6 ± 0.2	92.9	566.0 ± 65.2	98.1
3.5 µg mL^−1^ PS-NH_2_	56	254.5 ± 56.9 **	86.3	12.7 ± 9.8 ***	53.8	11.6 ± 11.5	12.0	1.3 ± 0.1 ***	76.0	537.8 ± 58.5 **	93.2
5 µg mL^−1^ PS-NH_2_	167	273.6 ± 83.7	92.8	8.6 ± 10.8 ***	36.4	24.7 ± 11.2	25.7	1.3 ± 0.2 ***	75.3	452.7 ± 113.4 *	78.4
7.5 µg mL^−1^ PS-NH_2_	141	259.0 ± 100.6	87.8	7.8 ± 9.7 ***	33.2	4.4 ± 4.3 ***	4.6	1.3 ± 0.2 ***	72.9	433.0 ± 97.0 *	75

## Data Availability

Data are contained within the article and Supplementary Materials.

## Supplementary Materials

The following supporting information can be downloaded at https://www.mdpi.com/article/10.3390/jox15010010/s1, Figures S1 and S2: FTIR spectrum of PS-NH_2_ vs. FTIR spectrum of polystyrene present in the libraries used for the match and FTIR spectrum of PS-NH_2_ vs. FTIR spectrum of non-functionalized fluorescent polystyrene nanoplastics (PS-NPs, 100 nm, Polysciences Europe GmbH, Eppelheim, Germany), respectively; Figure S3: Morphometric analysis of phenotypes induced in *Phallusia mammillata* embryos (A) with a chorion and (B) without a chorion exposed to PS-NH_2_. Radar charts summarize the following endpoints: ocellus (Oc) + otolith (Ot) area (μm^2^); Oc/Ot distance (μm); percentage of embryos with palps (%); trunk L/W (length/width) ratio; tail length (μm). All measurements were performed at 22 hpf. The values are normalized to the corresponding value of the same parameter in the control (stage 26) and presented as a percentage of the control value; Figure S4: Genotoxicity assay performed analyzing 7 hpf embryos (neurula stage). (A) Control embryo, (B) embryo with DNA aberration.
