# Supplementary material for: Nanoplastic-Induced Developmental Toxicity in Ascidians: Comparative Analysis of Chorionated and Dechorionated Phallusia mammillata Embryos
Source: J Xenobiot. 2025 Jan 10;15(1):10. doi: 10.3390/jox15010010 (PMC11755549; doi:10.3390/jox15010010)
Supplement: Supplementary file 1 [file jox-15-00010-s001.zip › Supplementary_Figure_S3.pdf]

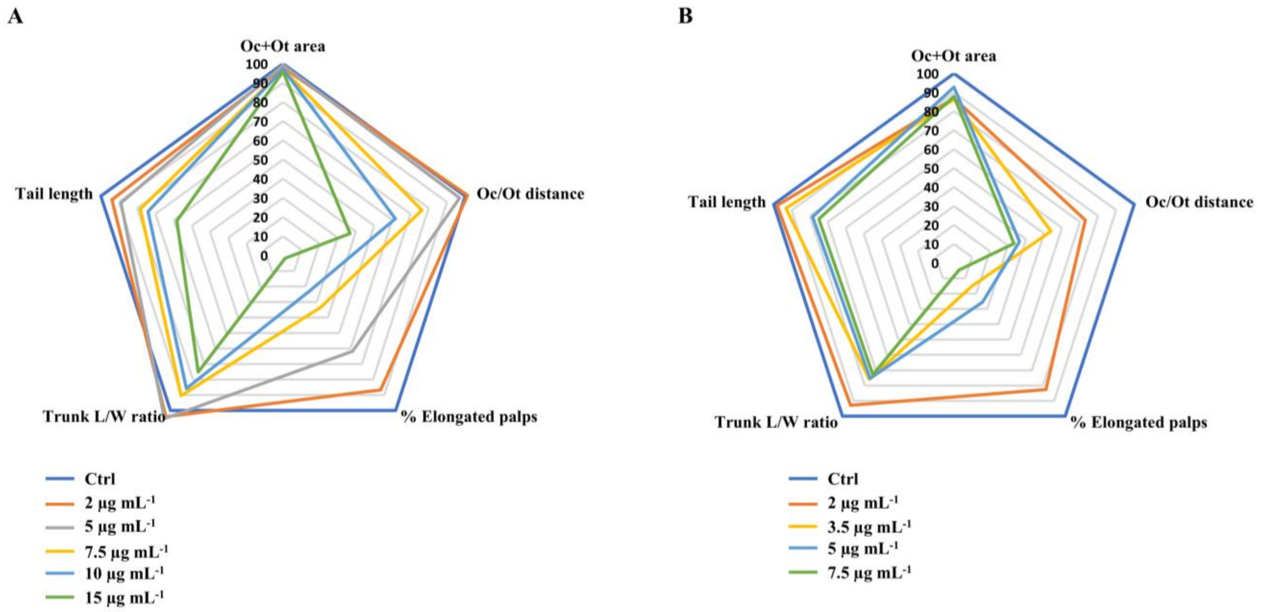

Figure S3. Morphometric analysis of phenotypes induced in Phallusia mammillata embryos (A) with chorion and (B) without chorion exposed to PS-NH2. Radar charts summarize the following endpoints: ocellus (Oc) + otolith (Ot) area (µm2); Oc/Ot distance (µm); percentage of embryos with palps (%); trunk L/W (length/width) ratio; tail length (µm). All measurements are performed at 22 hpf. The values are normalized to the corresponding value of the same parameter in the control (stage 26) and presented as a percentage of the control value.
