# Supplementary material for: Nanoplastic-Induced Developmental Toxicity in Ascidians: Comparative Analysis of Chorionated and Dechorionated Phallusia mammillata Embryos
Source: J Xenobiot. 2025 Jan 10;15(1):10. doi: 10.3390/jox15010010 (PMC11755549; doi:10.3390/jox15010010)
Supplement: Supplementary file 1 [file jox-15-00010-s001.zip › Supplementary_Figure_S4.pdf]

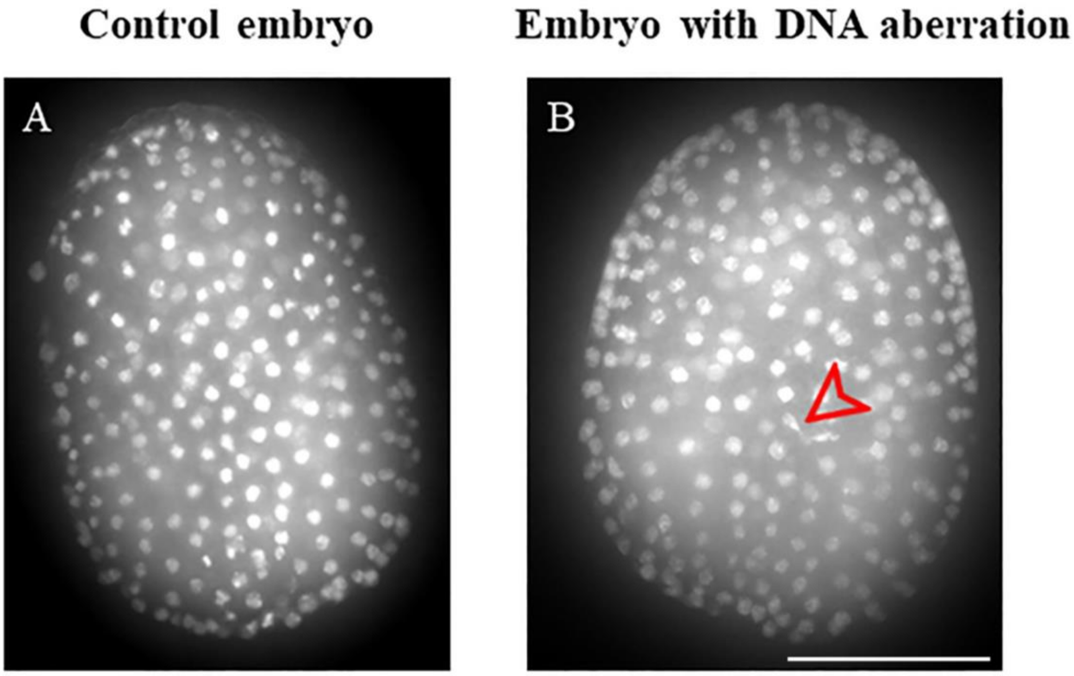

Figure S4. Genotoxicity assay performed analyzing 7 hpf embryos (neurula stage). (A) Control embryo, (B) Embryo with DNA aberration
