# Supplementary material for: Nanoplastic-Induced Developmental Toxicity in Ascidians: Comparative Analysis of Chorionated and Dechorionated Phallusia mammillata Embryos
Source: J Xenobiot. 2025 Jan 10;15(1):10. doi: 10.3390/jox15010010 (PMC11755549; doi:10.3390/jox15010010)
Supplement: Supplementary file 1 [file jox-15-00010-s001.zip › Supplementary_FTIR_analyses_Figure_S1_and_S2.pdf]

Characterization of polystyrene nanoparticles

For the purpose of Fourier Transform Infrared (FTIR) analysis, infrared measurements were conducted using the Nicolet i5 FTIR spectrometer (Thermo Fisher Scientific, MA, USA) equipped with a germanium tip-ATR crystal applied (4000- 675 cm). For all measurements, the number of scans were 32 and the spectral resolution was 4 cm-1. Data acquisition and spectral processing were performed with software (OMNIC v5.2, Nicolet, USA and Spectra, Thermo Fisher, USA, respectively). Polymer identification was performed by comparing the obtained spectrum with those of libraries with a matching value >80 %. The following libraries were used to identify the polymeric composition of each plastic item: HR Aldrich Polymers, HR Coatings Technology, HR Hummel Polymer and Additives, HR Industrial Coatings, HR Polymer Additives and Plasticizers, HR Rubber Compounding Materials, HR Spectra Polymers and Plasticizers, Hummel Polymer Sample Library and Polymer Laminate Films.

Title: PS NH2 Thu Jan 02 16:48:26 2025

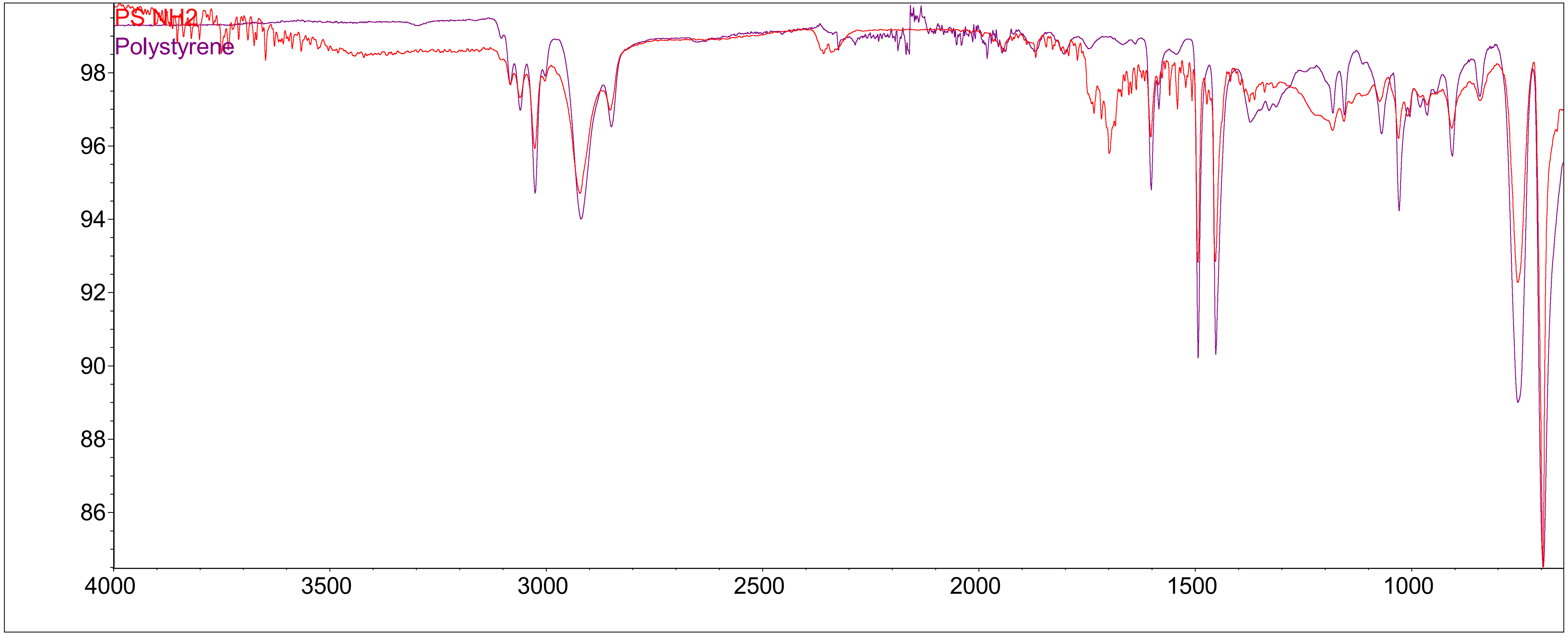

Collection time: Thu Jan 02 15:55:01 2025 (GMT+0[illegible]

No peak table for the selected spectrum!

The best match is very good,
but the second best match is also similar.

Spectrum: PS NH2
Region: 450.00-2600.00
Search type: Correlation
Hit List:

Index	Match	Compound name	Library
68	93.22	Polystyrene	HR Specta Polymers and Plasticizers by ATR
46	93.08	POLY(STYRENE), ATACTIC	Hummel Polymer Sample Library
82	90.20	Styrene/acrylonitrile copolymer 25%	HR Specta Polymers and Plasticizers by ATR

Figure S1. FTIR spectrum of PS-NH_2 vs FTIR spectrum of polystyrene present in the libraries used for the match.

Title: PS NH2 Thu Jan 02 17:02:35 2025

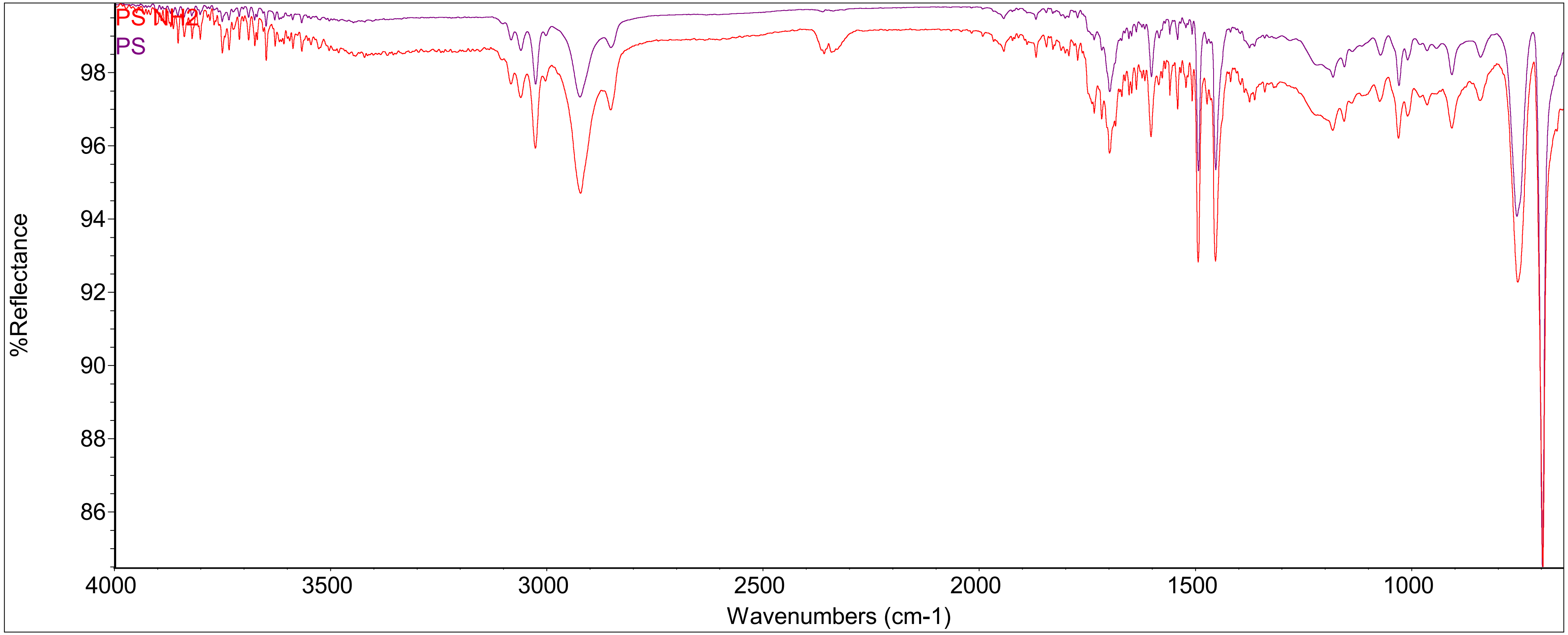

Collection time: Thu Jan 02 15:55:01 2025 (GMT+0[illegible]

No peak table for the selected spectrum!

The best match is very good,
but the second best match is also similar.

Spectrum: PS NH2
Region: 450.00-2600.00
Search type: Correlation
Hit List:

Index	Match	Compound name	Library
68	93.22	Polystyrene	HR Specta Polymers and Plasticizers by ATR
46	93.08	POLY(STYRENE), ATACTIC	Hummel Polymer Sample Library
82	90.20	Styrene/acrylonitrile copolymer 25%	HR Specta Polymers and Plasticizers by ATR

Figure S2. FTIR spectrum of PS-NH_2 *vs* FTIR spectrum non-functionalized fluorescent polystyrene nanoplastics (PS-NPs, 100 nm, Polysciences Europe GmbH, Eppelheim, Germany)
